# Interferon-β Produces Synergistic Combinatory Anti-Tumor Effects with Cisplatin or Pemetrexed on Mesothelioma Cells

**DOI:** 10.1371/journal.pone.0072709

**Published:** 2013-08-16

**Authors:** Quanhai Li, Kiyoko Kawamura, Shan Yang, Shinya Okamoto, Hiroshi Kobayashi, Yuji Tada, Ikuo Sekine, Yuichi Takiguchi, Masato Shingyouji, Koichiro Tatsumi, Hideaki Shimada, Kenzo Hiroshima, Masatoshi Tagawa

**Affiliations:** 1 Division of Pathology and Cell Therapy, Chiba Cancer Center Research Institute, Chiba, Japan; 2 Department of Molecular Biology and Oncology, Graduate School of Medicine, Chiba University, Chiba, Japan; 3 Department of Biochemistry, Graduate School of Pharmaceutical Science, Chiba University, Chiba, Japan; 4 Department of Respirology, Graduate School of Medicine, Chiba University, Chiba, Japan; 5 Department of Medical Oncology, Graduate School of Medicine, Chiba University, Chiba, Japan; 6 Division of Thoracic Diseases, Chiba Cancer Center, Chiba, Japan; 7 Department of Surgery, School of Medicine, Toho University, Tokyo, Japan; 8 Department of Pathology, Tokyo Women’s Medical University Yachiyo Medical Center, Yachiyo, Japan; Istituto Superiore di Sanità, Italy

## Abstract

Interferons (IFNs) have been tested for the therapeutic effects in various types of malignancy, but mechanisms of the anti-tumors effects and the differential biological activities among IFN members are dependent on respective cell types. In this study, we examined growth inhibitory activities of type I and III IFNs on 5 kinds of human mesothelioma cells bearing wild-type *p53* gene, and showed that type I IFNs but not type III IFNs decreased the cell viabilities. Moreover, growth inhibitory activities and up-regulated expression levels of the major histocompatibility complexes class I antigens were greater with IFN-β than with IFN-α treatments. Cell cycle analyses demonstrated that type I IFNs increased S- and G2/M-phase populations, and subsequently sub-G1-phase fractions. The cell cycle changes were also greater with IFN-β than IFN-α treatments, and these data collectively showed that IFN-β had stronger biological activities than IFN-α in mesothelioma. Type I IFNs-treated cells increased p53 expression and the phosphorylation levels, and activated apoptotic pathways. A combinatory use of IFN-β and cisplatin or pemetrexed, both of which are the current first-line chemotherapeutic agents for mesothelioma, produced synergistic anti-tumor effects, which were also evidenced by increased sub-G1-phase fractions. These data demonstrated firstly to our knowledge that IFN-β produced synergistic anti-tumor effects with cisplatin or pemetrexed on mesothelioma through up-regulated p53 expression.

## Introduction

Malignant mesothelioma, often linked with asbestos exposure, evokes serious social concerns in many countries, and the patient numbers in Western countries and newly industrializing economies will progressively increase in the next decades [1,2]. Mesothelioma spreads along the pleural cavity and is often resistant to conventional treatments. Extrapleural pneumonectomy is applicable to the cases only at the early phase, but the recurrence is common despite the radical operation procedures. The current therapeutic strategy for the majority of mesothelioma cases is primarily chemotherapy, and a combinatory use of cisplatin (CDDP) and pemetrexed (PEM) is the first-line regimen [3]. A median survival period with the regimen is however relatively short, about 12 months, and possible second-line anti-cancer agents have not yet been demonstrated.

Mesothelioma has an unusual molecular lesion linked with loss of tumor suppressor functions. The majority of mesothelioma has a deletion in the INK4A/ARF locus which encodes the *p14* ^*ARF*^ and the *p16* ^*INK4A*^ genes, but possesses the wild-type *p53* gene [4]. Deletion of p16^INK4A^ increases cyclin-dependent kinase 4/6 activities, which subsequently induces pRb phosphorylation and cell cycle progression. In contrast, deficiency of p14^ARF^ augments Mdm2 activities and consequently down-regulates p53 expression, which may render mesothelioma cells resistant to chemotherapeutic agents. Enhanced expression of p53 in mesothelioma is therefore a possible therapeutic strategy by inducing cell cycle arrest and apoptosis [5].

Interferons (IFNs) have anti-tumor effects by stimulating cell death and enforcing host immune systems. Three classes of IFNs have been identified, type I, II and III. Both type I and type III IFNs share similar biological activities including apoptosis induction, whereas type II IFN, IFN-γ, is primarily immune-stimulatory [6,7]. Type I IFNs, IFN-α and IFN-β, were well studied for the biological activities, and IFN-α but not IFN-β has been mainly tested for the anti-tumor actions in combination with anti-cancer agents in clinical settings. In contrast, type III IFNs, IFN-λs, have not been clinically tested for malignance and the precise mechanisms of type III IFNs-mediated apoptosis are not analyzed well [7,8]. As for mesothelioma, type I IFNs have not been rigorously studied for the therapeutic efficacy. There are only a few clinical studies on anti-tumor actions of IFN-α in combination with anti-cancer agents for mesothelioma [9–11], and combinatory effects of type I IFNs and PEM have not been examined. Recently, adenoviruses expressing the *IFN-β* gene were examined for the anti-tumor effects on mesothelioma in an animal model, and were clinically investigated for the safety and the therapeutic feasibility in mesothelioma patients [12,13]. Nevertheless, anti-tumor effects of recombinant type I IFNs in mesothelioma cells have not well studies particularly in terms of combination with the first-line chemotherapeutic agents. Moreover, differential biological activities between IFN-α and -β on mesothelioma remains uncharacterized.

A precise mechanism of IFN-mediated cell death also is unclear but Takaoka et al. demonstrated that type I IFNs up-regulated expression of the *p53* gene, suggesting a possible role of p53 in the type I IFN-mediated anti-tumor effects [14]. Nevertheless, type I IFNs produced apoptotic cell death even in *p53*-mutated tumors [15], which suggests p53 independent pathways in the IFNs-mediated cell death. In this study we compared anti-tumor effects of type I and type III IFNs with 5 kinds of *p53*-wild type mesothelioma cells, and investigated a possible up-regulation of p53 and combinatory effects of IFN with the first-line chemotherapeutic agents.

## Materials and Methods

### Cells

Human mesothelioma, NCI-H2452, NCI-H2052, NCI-H226, NCI-H28 and MSTO-211H cells, and mesothelium-derived Met-5A cells that were immortalized with the SV40 T antigen [16] were obtained from ATCC (Manassas, VA, USA). Human esophageal carcinoma T.Tn cells were from cell resource center for biomedical research, Tohoku University, Japan. They were cultured in RPMI-1640 medium supplemented with 10% fetal calf serum. All the mesothelioma cells used were defective of p14 and p16 expressions due to either loss of the transcription or deletion of the genomic DNA (Figure S1), and sequencing data confirmed that they possessed the wild-type *p53* gene.

### Reverse transcription-polymerase chain reaction (RT-PCR)

First-strand cDNA was synthesized with Superscript III reverse transcriptase (Invitrogen, Carlsbad, CA) and amplification of equal amounts of the cDNA was performed with the following primers and conditions: for the *IFNAR-1* gene, 5’-CTTTCAAGTTCAGTGGCTCCACGC-3’ (sense) and 5’-TCACAGGCGTGTTTCCAGACTG-3’ (anti-sense), and 10 sec at 94 ^°^C for denature/20 sec at 60 ^°^C for annealing/32 cycles; for the *IFNAR-2* gene, 5’-GAAGGTGGTTAAGAACTGTGC-3’ (sense) and 5’-CCC GCTGAATCCTTCTAGGACGG-3’ (anti-sense), and 10 sec at 94 ^°^C/20 sec at 56 ^°^C/31 cycles; for the *IL-28Rα* gene, 5’-GGGAACCAAGGAGCTGCTATG-3’ (sense) and 5’-TGGCACTGAGGCAGTGGTGTT-3’ (anti-sense), and 10 sec at 94 ^°^C/20 sec at 58 ^°^C/31 cycles; for the *IL-10Rβ* gene, 5’-TATTGGACCCCCTGGAAT-3’ (sense) and 5’-GTAAACGCACCACAGCAA-3’ (anti-sense), and 10 sec at 94 ^°^C/20 sec at 50 ^°^C/32 cycles; for the *GAPDH* gene, 5’-ACCACAGTCCATGCCATCAC-3’ (sense) and 5’-TCCACCACCCTGTTGCTGTA-3’ (anti-sense), and 15 sec at 94 ^°^C/15 sec at 60 ^°^C/25 cycles.

### Cell proliferation and viability test in vitro

Cells (1x10^3^/well) were seeded in 96-well plates and were cultured with IFN-α2a (IFN-α), IFN-β1a (IFN-β) (PBL Interferon Source, Piscataway, NJ, USA) or IFN-λ1 (R&D Systems, Minneapolis, MN, USA) at different doses. In a combinatory treatment, cells were treated with various concentrations of CDDP or PEM and with IFNs. Cell viabilities were assessed with a WST kit (Dojindo, Kumamoto, Japan) which detected the amounts of formazan produced from the WST-8 (2-(2-methoxy-4-nitrophenyl)-3-(4-nitrophenyl)-5-(2,4-disulfophenyl)-2H-tetrazolium) reagent with the absorbance at 450 nm (WST assay). The relative viability was calculated based on the absorbance without any treatments. Combinatory effects were examined with CalcuSyn software (Biosoft, Cambridge, UK). Combination index (CI) values at respective fractions affected (Fa), which showed relative suppression levels of cell viability, were calculated based on the WST assay. CI<1, CI=1 and CI>1 indicate synergistic, additive and antagonistic actions, respectively. Viable cell numbers were counted with the trypan blue dye exclusion test. The statistical analysis was performed with one way analysis of variance (ANOVA).

### Cell cycle analysis and cell surface staining

For cell cycle analysis, cells were fixed in ice-cold 70% ethanol, incubated with RNase (50 µg/ml) and stained with propidium iodide (PI) (50 µg/ml). For cell surface staining, cells were stained with fluorescein isothiocyanate (FITC)-conjugated anti-HLA-A, B,C antibody or FITC-conjugated isotype-matched control antibody (BD Biosciences, San Jose, CA, USA). The PI staining profiles and the FITC fluorescence intensity were analyzed with FACSCalibur and CellQuest software (BD Biosciences). The statistical analysis was performed with ANOVA.

### Western blot analysis

Cells were treated with IFNs and the cell lysate was subjected to sodium dodecyl sulfate polyacrylamide gel electrophoresis. The protein was transferred to a nylon filter and was hybridized with antibodies against p53 (Lab Vision, Fremont, CA, USA), phosphorylated p53 at serine (Ser) 15, Bax, cyclin E, caspase-3, cleaved caspase-3, caspase-8, cleaved caspase-8, caspase-9, cleaved caspase-9, FADD (Cell Signaling, Beverly, MA, USA), p21 (Santa Cruz Biotech, Santa Cruz, CA, USA), p27 (BD Biosciences), or actin (Sigma-Aldrich, St Louis, MO, USA). The membranes were developed with the ECL system (GE Healthcare, Buckinghamshire, UK).

## Results

### Expression of IFN receptor complexes

**Figure 1:**
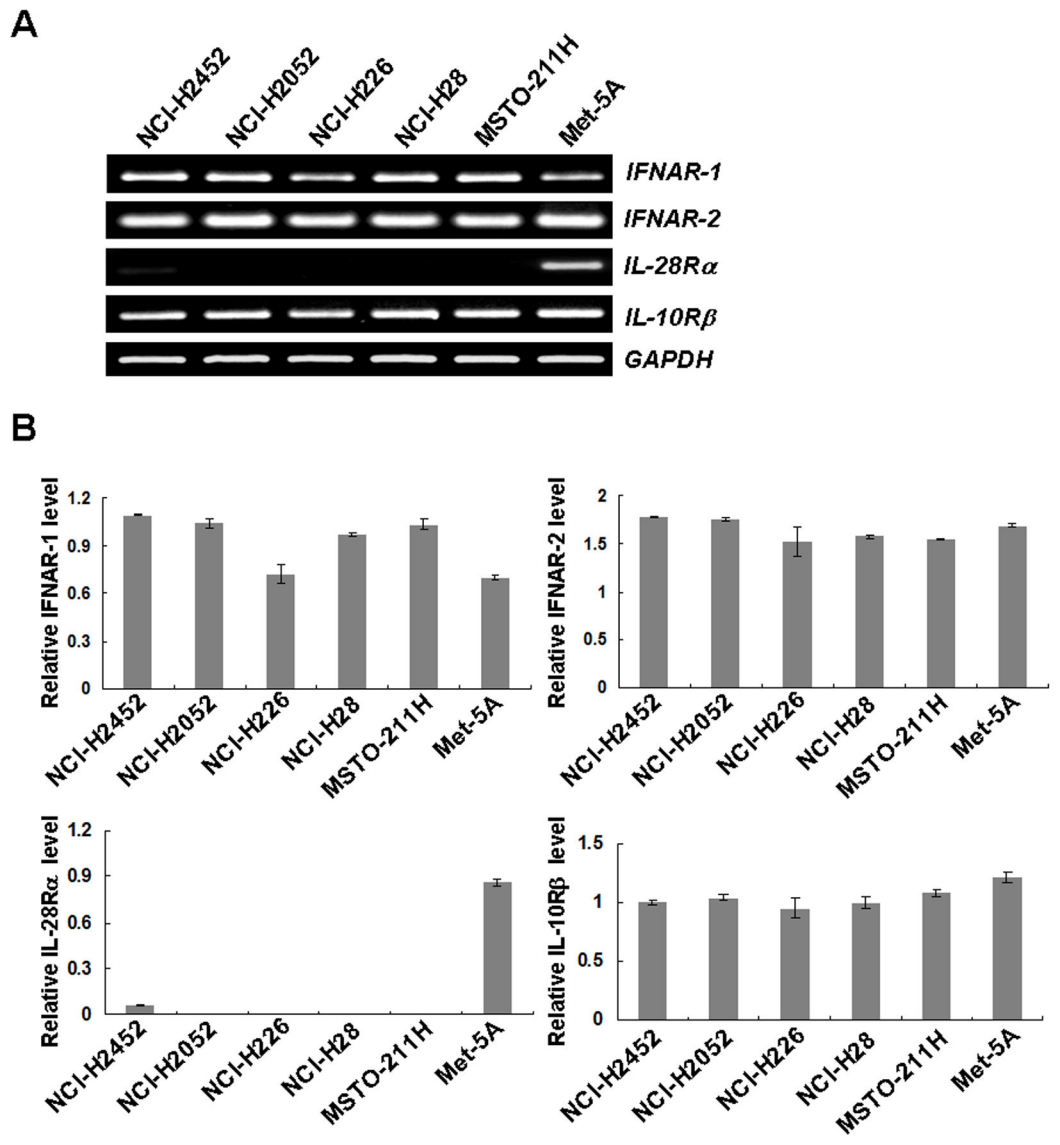
Type I and III IFNs receptors expression. (**A**) Expressions of *IFNAR-1*, *IFNAR-2*, *IL-28Rα* and *IL-10Rβ* genes were analyzed with RT-PCR. Representative data of 3 independent experiments and GAPDH is shown as a control. (**B**) Expression levels of *IFNAR-1*, *IFNAR-2*, *IL-28Rα* and *IL-10Rβ* genes were quantified with the ImageJ software (http://rsb.info.nih.gov/ij/features.html) and the respective relative intensities were determined based on the corresponding GAPDH expression level. Average intensities with SEs are shown (n=3).

We examined receptor expressions of type I and type III IFNs in 5 kinds of human mesothelioma and Met-5A cells with RT-PCR analyses (Figure 1). The type I IFN receptor complex consists of IFNAR-1 and IFNAR-2 molecules and all the cells expressed both receptor genes. In contrast, all the mesothelioma expressed only one of the heterodimeric type III receptor genes, the *IL-10Rβ*, but not the *IL-28Rα*. Immortalized Met-5A cells expressed both of the type III receptor genes. These data collectively suggested that the mesothelioma cells could respond to type I IFNs but not to type III IFNs, whereas Met-5 cells could response to both IFNs.

### Growth inhibitory action of IFNs on mesothelioma

**Figure 2:**
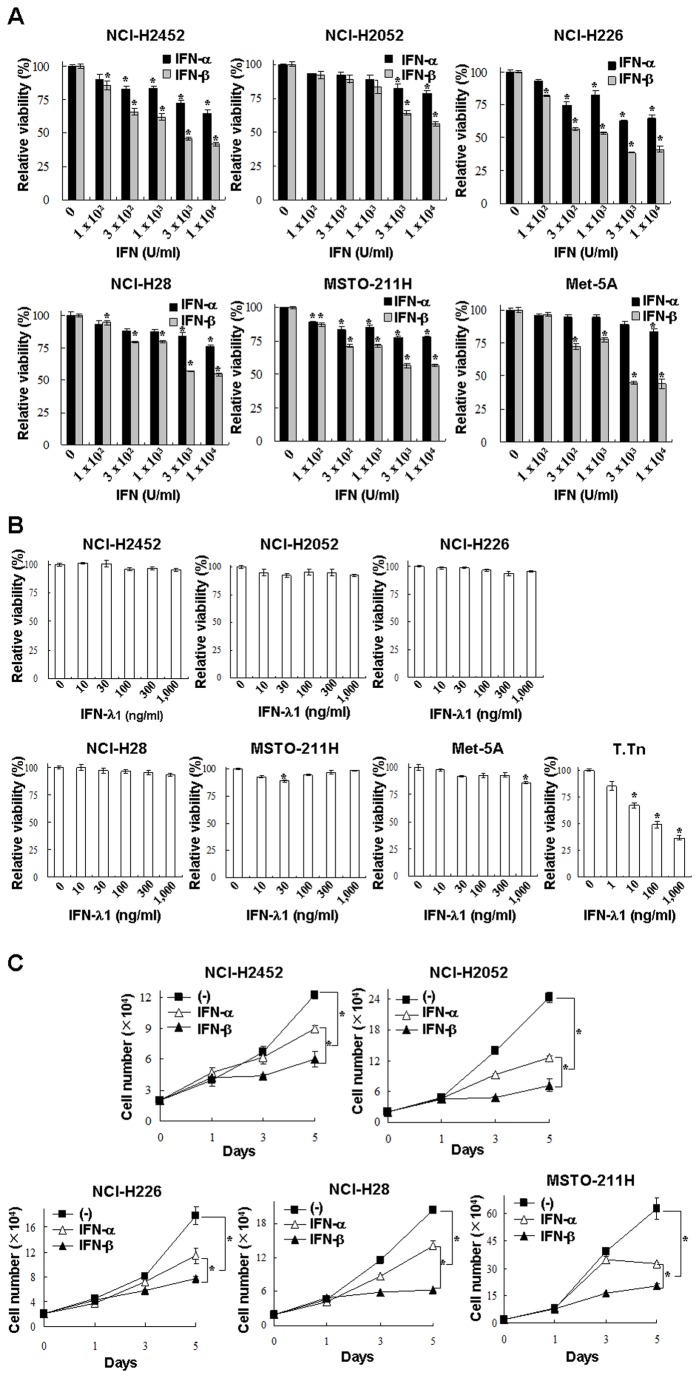
Growth inhibition by Type I and III IFNs. Cells were treated with various dose of IFN-α or -β (**A**), or IFN-λ1 (**B**) for 5 days, and the cell viabilities were measured with the WST assay. The relative viabilities were calculated based on the absorbance without any treatments. Means of triplicate samples and SE bars are shown (n=3). T.Tn cells, sensitive for IFN-λ1-mediated growth inhibition, were used as a positive control. *P < 0.01, comparing IFN-treated and untreated cells. (**C**) Cells were also cultured with or without IFN-α or -β (3,000 U/ml), and live cells numbers were determined with a dye exclusion test. Means and SE bars are shown (n=3), *P < 0.01.

We examined cytotoxic activities induced by IFNs with the panel of mesothelioma and Met-5A cells. IFN-α and IFN-β suppressed the viability in a dose-dependent manner, and the suppressive activities of IFN-β were greater with than those of IFN-α (Figure 2A). We confirmed the suppressive actions with Bonferroni multiple testing correction (Table S1). In contrast, IFN-λ1 did not produce growth inhibitory effects in mesothelioma cells, whereas human esophageal carcinoma T.Tn cells, which were positive for both IL-28Rα and IL-10β [17], were sensitive to IFN-λ1 (Figure 2B & Table S1). Growth of Met-5A cells was minimally suppressed with IFN-λ1 at a high dose. The differential susceptibility to type III IFNs, as found in T.Tn and Met-5A cells, suggested that sensitivity to IFN-λ1 was dependent on cell types even though they expressed type III receptor complexes [17]. IFN-α or -β-mediated growth inhibition was also demonstrated by counting live cells (Figure 2C & Table S1). The growth retardation caused by the same unit was greater with IFN-β than IFN-α in any of cells tested. These data demonstrated that type I but not type III IFNs suppressed proliferations of mesothelioma and that IFN-β had the inhibitory activities greater than IFN-α.

### Type I IFNs-mediated cell cycle changes

**Figure 3:**
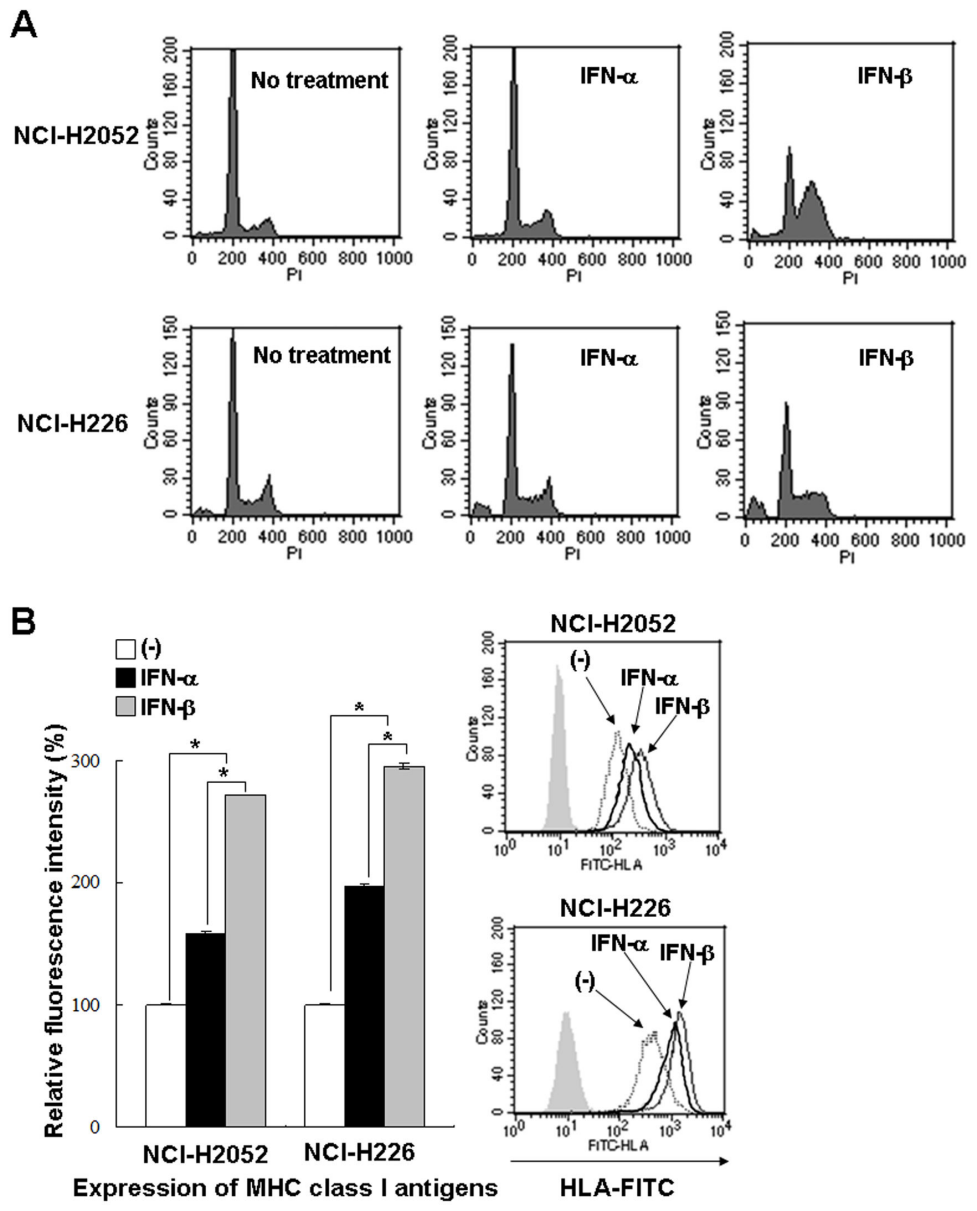
Compared biological activity between IFN-α and IFN-β. (**A**) NCI-H2052 and NCI-H226 cells were treated with or without IFNs (3,000 U/ml) and cultured for 72 hours. Cells were stained with PI and the cycle cycles were analyzed with flow cytometry. Representative data of 3 independent experiments are shown. (**B**) Cells were treated with or without type I IFNs (NCI-H2052; 300 U/ml, NCI-H226; 100 U/ml) and cultured for 24 hours. Mean fluorescence intensity of the MHC class I antigens analyzed with flow cytometry and the SE bars are also shown (n=3). *P < 0.01 Representative flow cytometrical data are also shown.

We compared cell cycle changes induced by I IFN-α and -β on mesothelioma and Met-5A cells (Table 1, representative data in Figure 3A). Type I IFNs-treated cells showed increased S- and G2/M-phase populations, and subsequently augmented sub-G1-phase fractions although these cell cycle alterations were dependent on respective cells used. The time-course data suggested that the increase at S-phase preceded that of G2/M-phase. The cell cycle changes, increased S- and G2/M-phase populations and then sub-G1 fractions, were greater with IFN-β treatments than with IFN-α treatments. Cell cycle changes of Met-5A were similar to mesothelioma cells except that Met-5A cells did not show increased sub-G1 populations. We also stained IFNs-treated cells with annexin V or PI, and found that the cells became annexin V-positive but not PI-positive (Table S2). These data suggested that mesothelioma cells were subjected to apoptosis with type I IFNs.

**Table 1:** Cell cycle distributions after type I IFNs treatments.

Cell			Treatment			Time (hr)		Cell cycle distribution (%)							
								sub-G1		G0/G1		S		G2/M	
NCI-H2452			(-)			48		1.5 ± 0.1		70.9 ± 0.3		10.5 ± 0.3		16.9 ± 0.5	
			IFN-α			48		1.9 ± 0.1		61.3 ± 0.3		14.0 ± 0.2^*^		22.7 ± 0.3^*^	
			IFN-β			48		3.2 ± 0.1^*^		45.8 ± 0.1		28.8 ± 0.6^**^		22.4 ± 0.5^*^	
			(-)			72		2.2 ± 0.1		66.1 ± 0.2		12.4 ± 0.1		19.5 ± 0.2	
			IFN-α			72		3.1 ± 0.2		55.3 ± 0.3		18.0 ± 0.2^*^		23.7 ± 0.2^*^	
			IFN-β			72		6.3 ± 0.8^*^		40.1 ± 0.4		28.8 ± 0.6^**^		24.9 ± 0.5^*^	
NCI-H2052			(-)			48		0.5 ± 0.1		77.9 ± 0.3		7.6 ± 0.2		14.1 ± 0.2	
			IFN-α			48		0.7 ± 0.1		71.9 ± 0.6		10.9 ± 0.4^*^		16.8 ± 0.2	
			IFN-β			48		1.1 ± 0.1		33.6 ± 1.5		36.8 ± 1.4^**^		29.3 ± 2.8^**^	
			(-)			72		0.9 ± 0.1		86.8 ± 0.1		4.2 ± 0.2		8.3 ± 0.1	
			IFN-α			72		0.7 ± 0.1		75.8 ± 0.3		8.7 ± 0.2^*^		15.1 ± 0.4^*^	
			IFN-β			72		3.2 ± 0.1^**^		33.6 ± 0.6		37.8 ± 0.6^**^		26.2 ± 1.3^**^	
NCI-H226			(-)			72		1.9 ± 0.2		73.2 ± 0.2		8.6 ± 0.3		16.1 ± 0.2	
			IFN-α			72		5.2 ± 0.2^**^		66.3 ± 0.3		12.1 ± 0.3^**^		15.9 ± 0.1	
			IFN-β			72		7.8 ± 1.2^**^		52.4 ± 1.2		20.5 ± 0.5^**^		18.5 ± 1.3	
MSTO-211H			(-)			48		3.9 ± 0.1		63.1 ± 0.2		12.5 ± 0.3		21.1 ± 0.2	
			IFN-α			48		5.9 ± 0.3		60.4 ± 0.5		15.1 ± 0.1^*^		19.4 ± 0.2	
			IFN-β			48		9.1 ± 0.3^*^		51.7 ± 0.7		16.6 ± 0.3^*^		23.1 ± 0.9	
			(-)			72		1.9 ± 0.1		83.7 ± 0.3		5.6 ± 0.2		8.9 ± 0.2	
			IFN-α			72		4.6 ± 0.2^*^		69.5 ± 0.3		9.8 ± 0.2^*^		16.8 ± 0.4^**^	
			IFN-β			72		16.7 ± 0.1^**^		44.8 ± 0.3		16.2 ± 0.1^**^		22.9 ± 0.3^**^	
NCI-H28			(-)			48		1.3 ± 0.1		65.4 ± 0.3		13.6 ± 0.2		19.6 ± 0.3	
			IFN-α			48		1.2 ± 0.1		70.5 ± 0.3		11.7 ± 0.2		16.6 ± 0.2	
			IFN-β			48		7.2 ± 0.1^**^		47.4 ± 0.6		26.5 ± 0.6^**^		18.9 ± 0.3	
			(-)			72		0.6 ± 0.1		73.2 ± 0.3		8.3 ± 0.3		17.9 ± 0.1	
			IFN-α			72		2.3 ± 0.1^**^		72.4 ± 0.3		9.8 ± 0.2		15.6 ± 0.5	
			IFN-β			72		12.4 ± 1.4^**^		50.8 ± 0.9		20.1 ± 0.8^**^		16.6 ± 1.4	
Met-5A			(-)			48		3.9 ± 0.9		68.1 ± 0.7		11.2 ± 0.6		16.5 ± 0.9	
			IFN-α			48		4.1 ± 0.7		61.9 ± 0.6		15.9 ± 0.2^*^		17.2 ± 0.5	
			IFN-β			48		5.0 ± 0.8		53.2 ± 1.9		22.9 ± 1.0^**^		17.7 ± 1.6	
			(-)			72		4.9 ± 0.7		72.5 ± 0.4		9.1 ± 0.4		13.1 ± 0.1	
			IFN-α			72		4.8 ± 0.3		61.0 ± 0.4		14.9 ± 0.1^**^		18.5 ± 0.6^*^	
			IFN-β			72		5.2 ± 0.8		51.9 ± 0.8		19.4 ± 0.3^**^		23.2 ± 0.8^**^	

Cells were treated with or without IFN-α or - β (3,000 U/ml) for the indicated periods. Cell cycle profiles were analyzed with flow cytometry, and the percent mean and SE of each fraction are shown (n=3).

^*^ *P* < 0.05, comparing IFN-α- or IFN-β-treated and untreated cells.

^**^ *P* < 0.01, comparing IFN-α- or IFN-β-treated and untreated cells.

We also examined up-regulated expression levels of class I molecules of the major histocompatibility complex (MHC) in mesothelioma cells (Figure 3B). IFN-α and -β increased the expression in NCI-H2052 and NCI-H226 cells, and the increased levels were greater with IFN-β than with IFN-α in both cells. In contrast, IFN-λ1 did not change the class I expression levels in these cells (data not shown). Compared activities of IFN-α and IFN-β on cell cycle changes and up-regulated expression of MHC class I molecules indicated that IFN-β had stronger biological actions than IFN-α.

### Activation of p53 and apoptotic pathways

**Figure 4:**
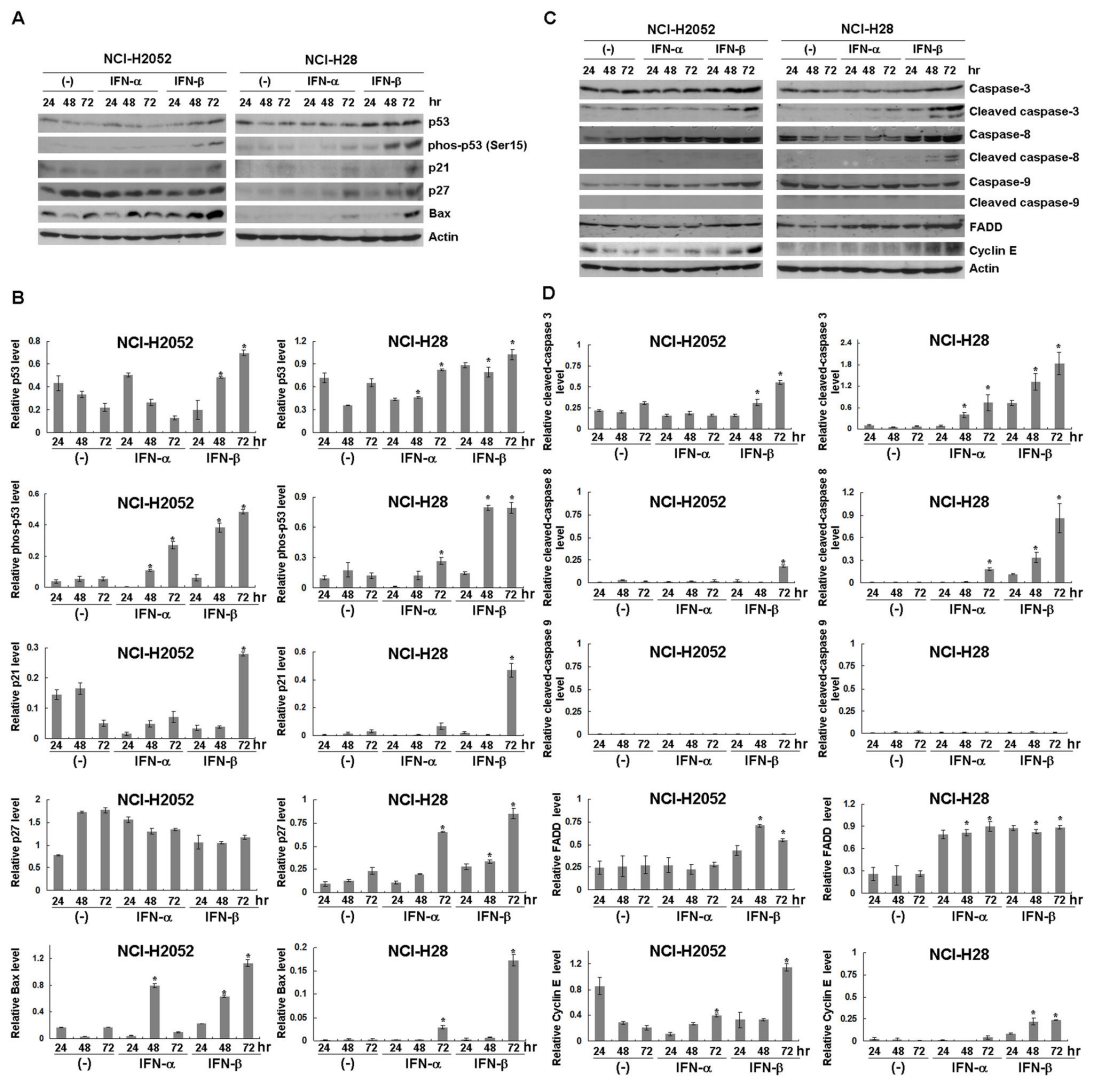
Expression of molecules involved in the p53 pathways, cell cycle and apoptosis. NCI-H2052 and NCI-H28 cells were treated with or without IFNs (3,000 U/ml) and cultured for the indicated time. Expression levels of (**A**) p53-, (**C**) cell cycle- and apoptosis-linked proteins were analyzed with Western blot analyses. Representative data of 3 independent experiments are shown and actin is used as a loading control. Relative expression levels of (**B**) p53 pathways-linked, (**D**) cell cycle-linked and apoptosis-linked molecules were quantified with the ImageJ software (http://rsb.info.nih.gov/ij/features.html). The relative intensity of each molecule was calculated based on (**B**) the corresponding actin, and (**D**) the uncleaved caspase (caspase-3, -8 and -9) or actin (FADD, cyclin E) expression level. (**B**) Relative phosphorylated p53 expression levels were determined based on the corresponding p53 expression level. Average intensities with SEs are shown (n=3). The statistical analysis was performed with ANOVA. *P < 0.05, comparing IFNs (3,000U/ml)-treated and untreated cells at 48 or 72 hr.

We further investigated possible p53 activation induced by the type I IFNs in 2 representative mesothelioma cells (Figure 4A & 4B). Cell cycle analyses after IFN-β treatments showed that NCI-H2052 cells increased S- and G2/M-phase fractions but less significantly augmented sub-G1 population, whereas NCI-H28 cells increased S-phase and then sub-G1 fractions (Table 1). The IFN-β treatment induced up-regulated p53 levels and the phosphorylation at Ser 15 residues, a marker of the p53 activation, in both NCI-H2052 and NCI-H28 cells, but these changes were undetectable or minimal in IFN-α-treated cells. IFN-β-treated NCI-H2052 and NCI-H28 cells showed up-regulated levels of p21 and Bax expression. The increased p21 expression was however undetectable in IFN-α-treated cells, and the up-regulated Bax expression was less significant in IFN-α-treated cells than in IFN-β-treated cells. Expression levels of p27 increased in NCI-H28 cells with either IFN-α or IFN-β treatment but those in NCI-H2052 cells remained unchanged.

We examined activations of caspases in IFNs-treated cells (Figure 4C & 4D). Cleavage of caspase-3 was augmented by IFN-β treatments in NCI-H2052 and NCI-H28 cells. Expression levels of cleaved and uncleaved caspase-8 were up-regulated in IFN-β-treated NCI-H28 cells and less significantly in IFN-β-treated NCI-H2052 cells. IFN-α treatments increased caspase-3 cleavage and slightly augmented caspase-8 cleavage only in NCI-H28 cells. Both IFN-β and IFN-α did not induce caspase-9 cleavage. Expression levels of FADD, an upstream molecule of the extrinsic pathway, increased in IFN-β-treated NCI-H28 and NCI-H2052 cells, and in IFN-α-treated NCI-H28 cells. These data showed that the activation was greater with IFN-β than with IFN-α and suggested that IFNs activated the extrinsic apoptotic pathway in mesothelioma. Expression levels of cyclin E, associated with G1- to S-phase progression [18,19], were also up-regulated in IFN-β-treated NCI-H28 and NCI-H2052 cells and in IFN-α-treated NCI-H2052 cells (Figure 4C & 4D).

### Combinatory effects of IFN-β and anti-cancer agents

**Figure 5:**
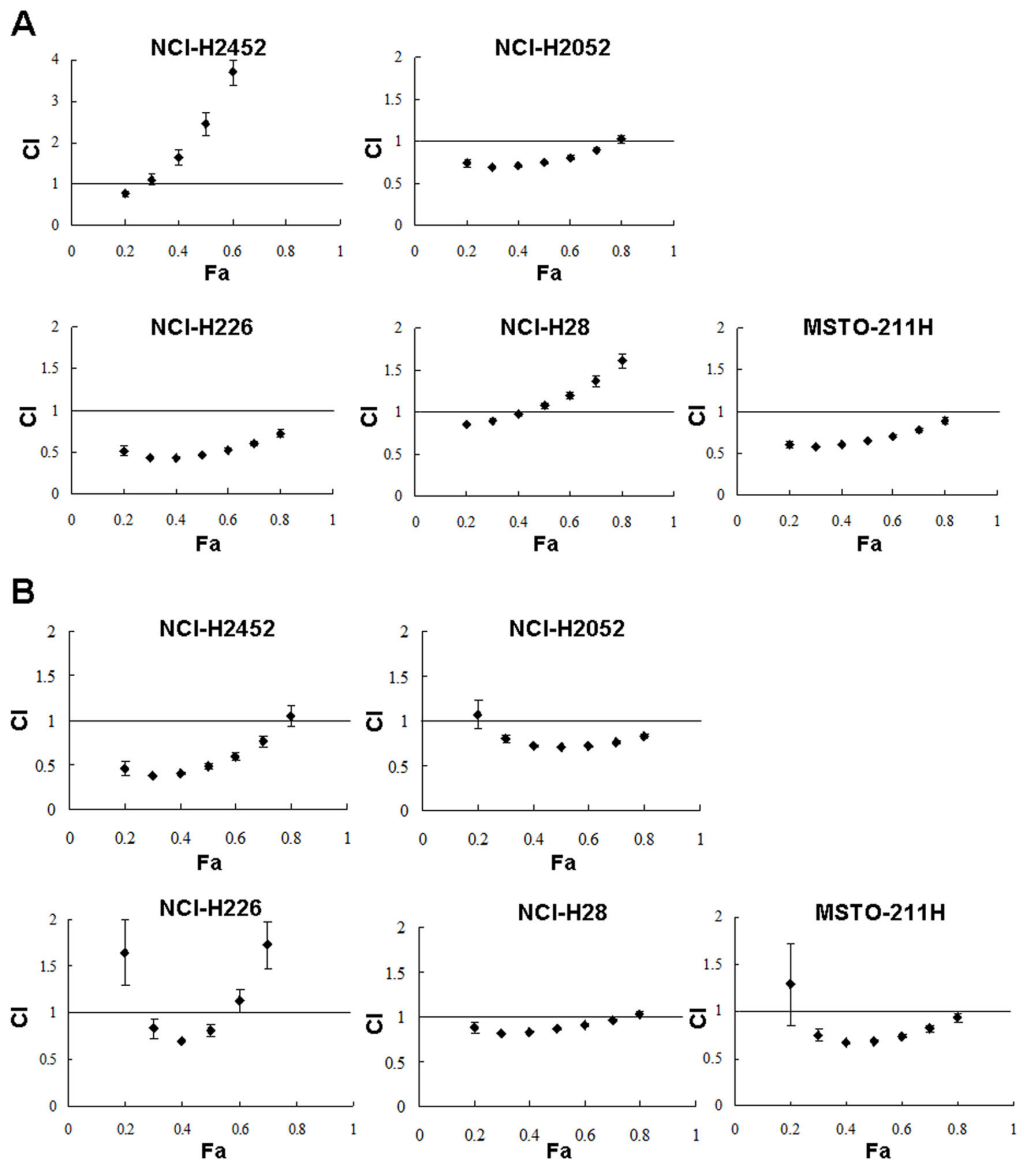
Combinatory effects of IFN-β and anti-cancer agents. Cells were treated either with (**A**) IFN-β (1,000U/ml) and CDDP (0, 0.3, 1, 3, 10 or 30 µM) or with (**B**) IFN-β (3,000 U/ml) and PEM (0, 3, 6, 12, 24 or 48 ng/ml), and were cultured for 4 days. CI values at respective Fa points are shown with SE bars (n=3).

We tested a possible combinatory cytotoxic activity produced by IFN-β and anti-cancer reagents, CDDP and PEM. Mesothelioma cells were treated with IFN-β and with CDDP or PEM at various concentrations, and the combinatory effects were examined by calculating CI values (Figure 5). The CI values at various Fa points showed that IFN-β and CDDP were synergistic in the inhibitory activity in most of the cells except NCI-H2452 cells at above 0.3 Fa points and NCI-H28 cells at above 0.4 Fa points (Figure 5A). Combination of IFN-β and PEM also produced synergistic effects in all the cells at Fa points between 0.3 and 0.7 except NCI-H226 cells at above 0.6 Fa points (Figure 5B). We also examined percentages of sub-G1-phase fractions induced by the combinatory treatment of IFN-β and CDDP or PEM in NCI-H2052 and NCI-H28 cells (Table 2). The sub-G1-phase populations were greater in cells treated with IFN-β and CDDP or PEM than those treated with IFN-β or the agents alone. In particular, the sub-G1-phase fractions in NCI-H2052 cells treated with IFN-β and CDDP or PEM were significantly great compared with those in I IFN-β-, CDDP- or PEN-treated cells. CDDP or PEM treatments increased S-phase fractions in NCI-H2052 and NCI-H28 cells, but the influence on G2/M-phase populations was variable.

**Table 2:** Cell cycle distributions by IFN-β and CDDP or PEM treatments.

Cell		IFN-β		Agent		Cell cycle distribution (%)							
						sub-G1		G0/G1		S		G2/M	
NCI-H2052		(-)		(-)		0.3 ± 0.1		85.8 ± 1.1		4.3 ± 0.2		9.7 ± 0.3	
		(+)		(-)		2.3 ± 0.1		53.4 ± 1.0		18.4 ± 0.9		26.5 ± 0.3	
		(-)		CDDP		1.5 ± 0.1		14.0 ± 0.6		19.7 ± 0.3		65.4 ± 0.3	
		(+)		CDDP		10.2 ± 0.1^**^		22.9 ± 0.4		53.9 ± 0.4		13.6 ± 0.2	
		(-)		PEM		9.1 ± 0.4		43.9 ± 0.3		38.6 ± 0.5		9.6 ± 0.4	
		(+)		PEM		22.2 ± 0.3^*^		49.8 ± 0.4		22.3 ± 0.6		6.6 ± 0.3	
NCI-H28		(-)		(-)		0.2 ± 0.1		69.8 ± 0.3		11.4 ± 0.1		18.9 ± 0.3	
		(+)		(-)		0.7 ± 0.1		61.6 ± 0.7		15.1 ± 0.1		23.4 ± 0.7	
		(-)		CDDP		11.2 ± 0.2		56.6± 1.0		19.3 ± 1.0		13.5 ± 0.3	
		(+)		CDDP		20.5 ± 0.4^*^		52.9 ± 0.6		15.6 ± 0.2		11.4 ± 0.2	
		(-)		PEM		3.0 ± 0.1		31.3 ± 0.1		44.4 ± 0.2		22.6 ± 0.3	
		(+)		PEM		8.1 ± 0.3^*^		37.5 ± 0.4		38.4 ± 0.5		17.0 ± 0.1	

Cells were incubated with or without IFN-β (3,000 U/ml) and also treated with or without CDDP (NCI-H2052; 3 µM, NCI-H28; 50 µM) or PEM (30 ng/ml) for 96 hr (NCI-H2052) or 72 hr (NCI-H28). Cell cycle profiles were analyzed with flow cytometry, and the percent mean and SE of each fraction are shown (n=3).

^*^ *P* < 0.05, comparing between IFN-β with CDDP- or PEM-treated cells and corresponding IFN-β-treated alone, or CDDP- or PEM-treated alone populations.

^**^ *P* < 0.01, comparing between IFN-β with CDDP-treated cells and corresponding IFN-β-treated alone or CDDP-treated alone populations.

## Discussion

In this study, we investigated cytotoxic effects of type I and type III IFNs and compared the biological activities between IFN-α and IFN-β. Moreover, we showed firstly to our knowledge combinatory cytotoxicity of IFN-β with the first-line anti-cancer agents for mesothelioma.

Type III IFNs was recently shown to produce anti-proliferative actions in a number of cancer cells [7,17], but the present study demonstrated that IFN-λ1 did not inhibit growth of mesothelioma cells due to the lack of IL-28Rα expression. Previous studies showed that expressions of type I IFN receptor complexes and IL-10Rβ were ubiquitous but that of IL-28Rα could be restricted in a tissue-specific manner [7]. Interestingly, Met-5A cells of mesothelium origin were positive for IL-28Rα in contrast to mesothelioma cells, suggesting that IL-28Rα expression was activated by a process of immortalization due to expressed SV40 T antigen or was lost during tumorigenesis of mesothelial cells. Met-5A cells were relatively insensitive to type III IFNs despite being positive for both of the receptor molecules. The sensitivity to type III IFNs was therefore not completely attributable to the receptor expression. In fact our previous study showed that 9 esophageal carcinoma cell lines expressed both the IL-28α and IL-10β receptors, but the IFN-λ1-mediated growth inhibition was observed only in some of the cell lines [17,20]. Type III IFNs-mediated growth suppression is thus dependent on cell types as well as IL-28Rα expression. Biomarkers to detect the growth inhibition are thereby required in the case of a possible clinical application with type III IFNs in future.

We also showed that IFN-β produced greater biological activities than IFN-α, which was evidenced by growth inhibitory actions, up-regulated expression levels of the MHC class I molecules and cell cycle changes. The mechanism underlying the greater biological functions of IFN-β than that of IFN-α is not well understood, but the differential binding affinity of both IFNs to type I IFN receptors and greater stability of IFN-β could be possible reasons [21].

Cell cycle analyses demonstrated that IFN-α and IFN-β treatments increased S- and G2/M-phase fractions, and then sub-G1-phase populations. Susceptibility of IFNs-mediated cell cycle changes was different among the mesothelioma cells tested. Interestingly, Met-5A cells were relatively resistant to increase of sub-G1-phase fractions although they showed increased S- and G2/M-phase populations. The lack of cell death in type I IFNs-treated Met-5A cells can be associated with the relative insensitivity to type III IFNs because Met-5A cells showed low proliferation activity compared with mesothelioma cells. IFNs up-regulated p53 and the phosphorylation levels, and subsequently induced p21 in mesothelioma cells, whereas IFNs did not induce G0/G1-phase arrests. IFN-β rather up-regulated cyclin E expressions which were linked with cell cycle shift to S-phase. Several lines of initial studies however showed that type I IFN-treated tumor cells became arrest at G0/G1-phase [22]. These data collective suggested that multiple factors were involved in the type I IFNs-induced cell cycle changes. Western blot analyses showed that IFN-β treatments increased caspase-3 cleavages in NCI-H28 cells. IFN-β also induced cleavage of caspase-8 and augmented FADD expression, but did not influence caspase-9 cleavage, indicating that the extrinsic death receptor-linked pathways play a role in the apoptosis. In contrast, previous studies showed that both the extrinsic and the intrinsic pathways-mediated apoptosis were induced by type I IFNs [23], and therefore further investigations are required for understanding the mechanism underlying preferential activation of extrinsic pathways in mesothelioma.

The present study showed that type I IFNs activated the p53 pathways in mesothelioma cells bearing the wild-type *p53* gene. The activated pathways were evidenced by phosphorylated p53 at Ser 15 residue and increased p53 levels together with augmented expression of p53 target molecules, p21, p27 and Bax. Takaoka et al. showed that IFN-β induced p53 protein expression through up-regulated p53 mRNA in fibroblasts, but did not induce the p53 phosphorylation [14]. In contrast, our present study demonstrated that IFN-β treatment itself induced p53 phosphorylation. Our previous study also showed that CDDP treatments increased endogenous p53 levels in mesothelioma cells, and the susceptibility to CDDP was augmented by forced p53 expression [5]. The synergistic combinatory effects between IFN-β and CDDP demonstrated in the present study can be due to augmented p53 activation. In contrast, a possible role of p53 in PEM-mediated cytotoxicity is controversial [24,25] although PEM induced DNA damages and apoptosis [26]. The present data of synergistic combinatory cytotoxicity between IFN-β and PEM rather suggested that PEM-mediated cytotoxicity could be enhanced by IFN-β-mediated p53 up-regulation. Several clinical trials of recombinant IFN-α with an anti-cancer agent have been conducted for mesothelioma [9–11], but possible combinatory effects with IFN-β and CDDP or PEM was not yet demonstrated in any of previous preclinical studies.

Sandoval et al. demonstrated that p14^ARF^ was required for type I IFNs-mediated apoptosis and for p53 up-regulation, and that IFN-β induced apoptosis even in a p53-defective state as long as p14 was intact [27]. The present study however demonstrated that IFN-β induced apoptosis and augmented p53 expression in NCI-H2052 and NCI-H28 cells which were defective of the *p14* ^*ARF*^ gene (Figure S1). Moreover, Met-5A cells, with *p14* ^*ARF*^ transcripts but loss of p53 functions, did not increase the sub-G1-phase populations with IFN-β treatments. It is currently unknown as to the p14 involvement in the type I IFNs-mediated apoptosis, but difference of genetic backgrounds, such as defective 16 expression and aberrant signal pathways often found in mesothelioma, can be responsible [28].

In summary, we demonstrated that IFN-β produced greater biological functions than IFN-α in mesothelioma, activated the p53 pathways and enhanced the anti-tumor effects of the first-line chemotherapeutic agents. Combinatory effects of IFN-β with the current first-line agents need to be re-evaluated since previous clinical studies for mesothelioma used IFN-α and non-current chemotherapeutic agents. The present study also suggests a possible clinical trial of intrapleural injections of adenoviruses expressing the *IFN-β* gene and systemic administrations of the first-line agents. Several clinical phase I studies with IFN-β-producing adenovirus vectors demonstrated feasibility of such gene therapy [12] but none of the clinical studies in combination with chemotherapy has been performed. Gene medicine with chemotherapeutic agents is one of the clinical trials to be examined in mesothelioma patients.

## Supporting Information

Figure S1 **Lack of the INK4A/ARF locus in mesothelioma.** (**A**) PCR to detect the p14^*ARF*^ gene consisting of exon 1β, 2 and 3, and the p16^*INK4A*^ gene consisting of exon 1α, 2 and 3. Both the p14^*ARF*^ and the p16^*INK4A*^ genes share the same exons 2 and 3. (**B**) RT-PCR to detect the p14^*ARF*^ and the p16^*INK4A*^ transcripts with primers designed between the exon 1β and the exon 2 for the p14^*ARF*^ and between the exon 1α to the exon 2 for the p16^*INK4A*^ gene. The data indicated that mesothelioma cells used in the present study did not express the p14^*ARF*^ or the p16^*INK4A*^ gene.
(TIF)

Table S1 **All the experimental data of Figure 2A, 2B and 2C were analyzed with Bonferroni test at SPSS 13.0 version.** The list shows only experiments with statistical significance (*P* < 0.05) and those without the significance (*P* above 0.05) were excluded. ^a^IFN-α and IFN-β; U/ml, IFN-λ; ng/ml. ^b^Live cell numbers cultured for 5 days were analyzed in the Figure 2C data.
(DOCX)

Table S2 **NCI-H28 cells were untreated or treated with IFN-α or IFN-β (3,000 U/ml) for 5 days.** The cells were then stained with PI and annexin V (Tali apoptosis kit, Life Technologies, Carlsbad, CA, USA) and were analyzed with Tali image-based cytometer (Life Technologies). The mean of stained cells percentage and the SE are shown (n = 3). The statistical analysis was performed with ANOVA. * *P* < 0.05, comparing IFN-α- or IFN-β-treated and untreated cells.
(DOCX)
