# Supplementary figures and images for: Interferon-β Produces Synergistic Combinatory Anti-Tumor Effects with Cisplatin or Pemetrexed on Mesothelioma Cells
Source: PLoS One. 2013 Aug 16;8(8):e72709. doi: 10.1371/journal.pone.0072709 (PMC3745385; doi:10.1371/journal.pone.0072709)

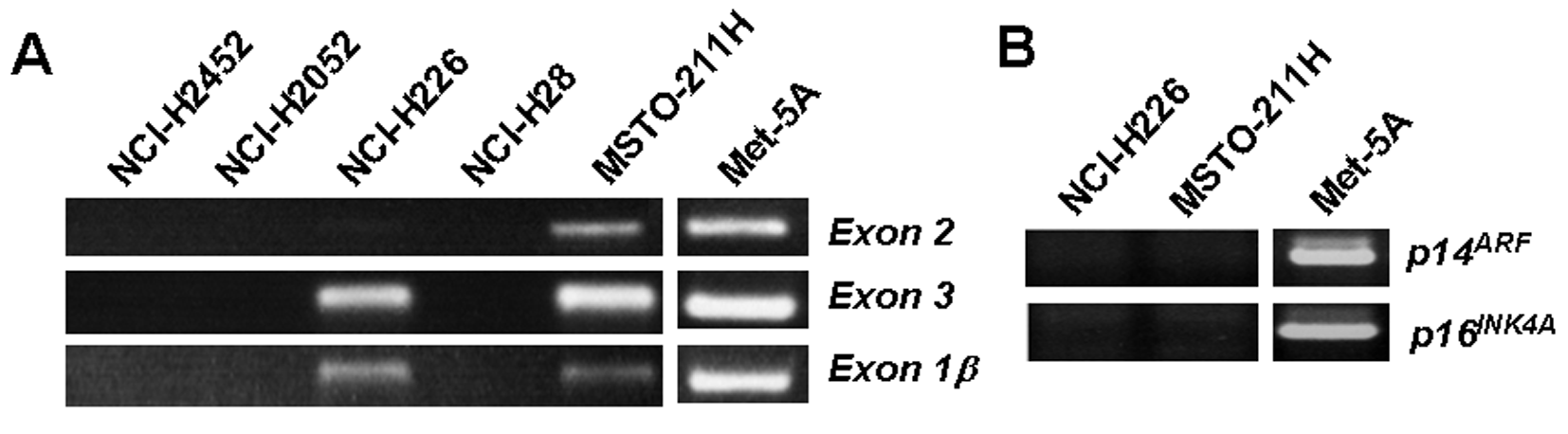

Supplement: Figure S1 — Lack of the INK4A/ARF locus in mesothelioma. (A) PCR to detect the p14ARF gene consisting of exon 1β, 2 and 3, and the p16INK4A gene consisting of exon 1α, 2 and 3. Both the p14ARF and the p16INK4A genes share the same exons 2 and 3. (B) RT-PCR to detect the p14ARF and the p16INK4A transcripts with primers designed between the exon 1β and the exon 2 for the p14ARF and between the exon 1α to the exon 2 for the p16INK4A gene. The data indicated that mesothelioma cells used in the present study did not express the p14ARF or the p16INK4A gene. (TIF) [file pone.0072709.s001.tif]
