# Supplementary material for: Interferon-β Produces Synergistic Combinatory Anti-Tumor Effects with Cisplatin or Pemetrexed on Mesothelioma Cells
Source: PLoS One. 2013 Aug 16;8(8):e72709. doi: 10.1371/journal.pone.0072709 (PMC3745385; doi:10.1371/journal.pone.0072709)
Supplement: Table S1 — All the experimental data of Figure 2A, 2B and 2C were analyzed with Bonferroni test at SPSS 13.0 version. The list shows only experiments with statistical significance (P < 0.05) and those without the significance (P above 0.05) were excluded. aIFN-α and IFN-β; U/ml, IFN-λ; ng/ml. bLive cell numbers cultured for 5 days were analyzed in the Figure 2C data. (DOCX) [file pone.0072709.s002.docx]

**Table S1.**Statistical analysis data in Figure 2 with Bonferroni correction.

| Cell | Treatment | Dose tested^a^ | Figure^b^ | *F* value | *P* value |
| --- | --- | --- | --- | --- | --- |
| NCI-H2452 | IFN-α | 0, 1x10^3^, 1x10^4^ | Figure 2A | 104.440 | Less than 0.002 |
|  | IFN-β | 0, 1x10^2^, 3x10^2^, 3x10^3^ | Figure 2A | 107.970 | Less than 0.011 |
|  | IFN-α,  IFN-β | 0, 3x10^3^ | Figure 2C | 106.094 | Less than 0.001 |
| NCI-H2052 | IFN-β | 0, 1x10^3^, 3x10^3^ | Figure 2A | 35.091 | Less than 0.024 |
|  | IFN-α,  IFN-β | 0, 3x10^3^ | Figure 2C | 283.196 | Less than 0.001 |
| NCI-H226 | IFN-α | 0, 3x10^2^, 3x10^3^ | Figure 2A | 108.304 | Less than 0.011 |
|  | IFN-β | 0, 1x10^2^, 3x10^2^, 3x10^3^ | Figure 2A | 1068.277 | Less than 0.001 |
|  | IFN-α,  IFN-β | 0, 3x10^3^ | Figure 2C | 63.789 | Less than 0.020 |
| NCI-H28 | IFN-α | 0, 3x10^2^, 1x10^4^ | Figure 2A | 29.845 | Less than 0.027 |
|  | IFN-β | 0, 1x10^2^, 3x10^2^, 3x10^3^ | Figure 2A | 296.040 | Less than 0.044 |
|  | IFN-α,  IFN-β | 0, 3x10^3^ | Figure 2C | 517.459 | Less than 0.001 |
| MSTO-211H | IFN-α | 0, 1x10^3^, 3x10^3^ | Figure 2A | 108.698 | Less than 0.007 |
|  | IFN-β | 0, 1x10^2^, 3x10^2^, 3x10^3^ | Figure 2A | 232.112 | Less than 0.001 |
|  | IFN-α,  IFN-β | 0, 3x10^3^ | Figure 2C | 112.694 | Less than 0.016 |
| Met-5A | IFN-β | 0, 3x10^2^, 3x10^3^ | Figure 2A | 201.640 | Less than 0.001 |
| T.Tn | IFN-λ | 0, 1, 10, 1x10^2^ | Figure 2B | 66.814 | Less than 0.031 |
