# Supplementary material for: Interferon-β Produces Synergistic Combinatory Anti-Tumor Effects with Cisplatin or Pemetrexed on Mesothelioma Cells
Source: PLoS One. 2013 Aug 16;8(8):e72709. doi: 10.1371/journal.pone.0072709 (PMC3745385; doi:10.1371/journal.pone.0072709)
Supplement: Table S2 — NCI-H28 cells were untreated or treated with IFN-α or IFN-β (3,000 U/ml) for 5 days. The cells were then stained with PI and annexin V (Tali apoptosis kit, Life Technologies, Carlsbad, CA, USA) and were analyzed with Tali image-based cytometer (Life Technologies). The mean of stained cells percentage and the SE are shown (n = 3). The statistical analysis was performed with ANOVA. * P < 0.05, comparing IFN-α- or IFN-β-treated and untreated cells. (DOCX) [file pone.0072709.s003.docx]

**Table S2.** PI and annexin V stained- populations in IFNs-treated cells.

| Treatment | Percentage of cells (%) (Average ± SE) | | |
| --- | --- | --- | --- |
|  | PI^-^/Annexin V^-^ | PI^+^/Annexin V^-^ | PI^-^/Annexin V^+^ |
| (-) | 86.3 ± 0.85 | 7.0 ± 0.58 | 6.5 ± 0.50 |
| IFN-α | 81.3 ± 0.24 | 4.3 ± 0.25 | 14.5 ± 0.29^*^ |
| IFN-β | 68.8 ± 1.44 | 5.0 ± 0.01 | 26.0 ± 1.23^*^ |
